# Effect of Constitution on Mass of Individual Organs and Their Association with Metabolic Rate in Humans—A Detailed View on Allometric Scaling

**DOI:** 10.1371/journal.pone.0022732

**Published:** 2011-07-26

**Authors:** Manfred J. Müller, Dirk Langemann, Isabel Gehrke, Wiebke Later, Martin Heller, Claus C. Glüer, Steven B. Heymsfield, Anja Bosy-Westphal

**Affiliations:** 1 Institut für Humanernährung und Lebensmittelkunde, Christian-Albrechts-Universität zu Kiel, Kiel, Germany; 2 Institute of Computational Mathematics, Technical University Braunschweig, Braunschweig, Germany; 3 Klinik für Diagnostische Radiologie, Universitätsklinikum Schleswig Holstein, Kiel, Germany; 4 Pennington Biomedical Research Center, Baton Rouge, Louisiana, United States of America; Indiana University, United States of America

## Abstract

Resting energy expenditure (REE)-power relationships result from multiple underlying factors including weight and height. In addition, detailed body composition, including fat free mass (FFM) and its components, skeletal muscle mass and internal organs with high metabolic rates (i.e. brain, heart, liver, kidneys), are major determinants of REE. Since the mass of individual organs scales to height as well as to weight (and, thus, to constitution), the variance in these associations may also add to the variance in REE. Here we address body composition (measured by magnetic resonance imaging) and REE (assessed by indirect calorimetry) in a group of 330 healthy volunteers differing with respect to age (17–78 years), sex (61% female) and BMI (15.9–47.8 kg/m^2^). Using three dimensional data interpolation we found that the inter-individual variance related to scaling of organ mass to height and weight and, thus, the constitution-related variances in either FFM (model 1) or kidneys, muscle, brain and liver (model 2) explained up to 43% of the inter-individual variance in REE. These data are the first evidence that constitution adds to the complexity of REE. Since organs scale differently as weight as well as height the “fit” of organ masses within constitution should be considered as a further trait.

## Introduction

Empirically-derived equations to predict resting energy expenditure (REE) in humans include weight (or a measure of metabolically active body mass), height, age and sex as determinants. According to Max Kleiber's inter-species analysis published in 1932, REE scales as weight^0.75^ (i.e., the ¾ power law [1]). More recent within-species data in humans reveal REE scaling to weight with powers of 0.64 to 0.73, suggesting some population-specific or between-studies diversities [2]. In addition to weight, REE also scales as height^1.5^ and REE per weight (i.e., mass-specific metabolic rate) scales as height^−0.5^ [2]. Obviously, we are faced with multiple scaling relationships. Detailed body composition analysis may add to our understanding of the combined effects of weight and height on REE.

REE-power relationships are the result of multiple underlying factors. Internal mechanisms include anatomical aspects of the body (i.e., body weight, it's composition, cell size and cell number). REE is a complex feature resulting from the mass and the mix of energy-demanding components within a body as well as their functions (e.g. basic maintenance processes like protein synthesis and ion transport by membrane pumps in different cells). However, the mechanistic basis of the relationship between metabolic rate and body weight is not fully explained. Up to 80% of the between subject variability in REE is explained by fat free mass (FFM [3]). Whole body REE can be further expressed as the sum of energy expended by individual organs and tissues within FFM [3]–[11]. Including the mass of individual organs into a regression analysis increased the explained inter-individual variance in REE to 86% [3]. Accordingly, modeling REE assuming constant organ and tissue metabolic rates [4]–[11], only small differences were found between measured and modeled REE (i.e., about 100 kJ/d) which did not support a mass-dependency of organ metabolic rates. The data thus suggests that the specific metabolic rate in humans with larger body mass is similar to those with smaller body masses [11].

Regarding detailed body composition measurements as assessed by magnetic resonance imaging *in vivo* our previous studies also showed that FFM, skeletal muscle mass and liver mass all scaled to height [2], [12], [13], suggesting that effects of stature on REE are partly explained by relationships to organ mass. The above mentioned increases in REE with height are then explained by parallel increases of height and weight (and thus, FFM and the mass of metabolically active organ mass) which all differ with respect to their scaling to height and weight and, thus, their contribution to the increase in REE with increasing height and weight. Since individual organs have different metabolic rates but also have distinctive and multiple scaling relationships (e.g., liver scaled to height with powers of about 2, whereas brain scaled to height with a power of 0.83) there is a need for an additional framework to predict REE.

Weight reflects the integrated effects of diet, activity, and genetic contributions to organ and tissue mass in the individual subject. Part of the between-individual variability in the relations between weight and body composition can be accounted for by between-individual differences in height. These multiple contributions lead to wide variation between individuals in the organ-tissue make up of body weight; we refer to this variation, after considering weight and height, as an individual's constitution. Individuals may not only differ in height and weight but also with respect to their constitution. Organs that are relatively small for individual weight and height may have a higher specific metabolic rate whereas relatively larger organs may have lower specific metabolic rates. Thus, besides the mass of organs, constitution may further add to the variance in specific metabolic rates.

Accordingly, we examined the scaling of organ mass to height and to weight in a large group of healthy subjects to assess whether inter-individual differences in organ mass per height and weight adds to the variance in REE. We also tried to address the question whether organ size relative to weight and height influences specific metabolic rates and thus adds to variance in REE.

## Results

### Descriptive presentation of data on body composition and resting energy expenditure

Descriptive data on our study population are presented in **Table 1**. Significant sex differences are present in age, height, weight, waist circumference, fat, muscle, organ mass (except for liver) and REE. Men were more frequently overweight while women were more frequently obese.

**Table 1:** Characterisation of the study population: anthropometric data, prevalence of overweight and obesity, body composition and resting energy expenditure (means ±SD, range) (n = 262).

	men	*SEE*	*n*	women	*SEE*	*n*
**age** (years)	45.5±15.2* (18–72)	*1.5*	*106*	38.6±13.8 (22–78)	*1.1*	*156*
**height** (m)	1.78±0.06* (1.61–1.95)	*0.006*	*106*	1.67±0.07 (1.48–1.86)	*0.005*	*156*
**weight** (kg)	84.6±13.4* (58.2–120.5)	*1.3*	*106*	80.2±20.8 (44.0–136.6)	*1.7*	*156*
**BMI** (kg/m^2^)	26.5±3.7 (18.3–36.8)	*0.3*	*106*	28.4±6.8 (16.8–46.8)	*0.5*	*156*
**WC** (cm)	94.6±12.4* (68.0–126.0)	*1.2*	*106*	93.1 ±16.8 (65–131)	*1.3*	*156*
**Prevalence**						
**overweight (BMI ≥25-<30 kg/m^2^)**	45.3%		*106*	19.2%		*156*
**obesity (BMI ≥30 kg/m^2^)**	17.9%		*106*	39.1%		*156*
**FM_DXA_** (kg)	18.9±8.2* (4.3–43.7)	*0.8*	*104*	30.4±13.9 (4.2–68.7)	*1.1*	*156*
**FFM_DXA_** (kg)	65.8±7.5* (48.5–92.6)	*0.7*	*104*	49.8±8.6 (34.1–79.5)	*0.7*	*156*
**MM_DXA_** (kg)	32.0±3.9* (22.1–44.1)	*0.4*	*104*	23.6±5.0 (15.2–39.1)	*0.4*	*156*
**organ mass, MRI**						
** brain** (g)	1609±100* (1350–1882)	*0.009*	*105*	1448±93 (1245–1696)	*0.007*	*148*
** Heart** (g)	384±73* (233–625)	*0.007*	*104*	306±55 (177–543)	*0.005*	*149*
** liver** (g)	1774±311 (1087–2584)	*0.03*	*105*	1582±312 (980–2588)	*0.03*	*152*
** kidneys** (g)	329±58* (205–488)	*0.005*	*100*	293±75 (162–546)	*0.006*	*150*
** spleen** (g)	309±120* (86–670)	*0.01*	*105*	220±67 (82–561)	*0.007*	*152*
**REE** (MJ/d)	7.5±0.9* (5.7–10.1)	*0.08*	*106*	6.4±1.1 (4.3–10.2)	*0.08*	*155*
**REE adj** (MJ/d)	7.3±0.6* (5.0–9.0)	*0.05*	*104*	6.3±0.5 (4.5–8.5)	*0.04*	*155*

*p<0.01 for sex differences (t-Test).

BMI, body mass index; WC, waist circumference; DXA, Dual Energy X-ray Absorptiometry; FM, fat mass; FFM, fat-free mass; MM, muscle mass; MRI, Magnetic Resonance Imaging; REE, resting energy expenditure, REEadj, resting energy expenditure adjusted for FFM, SEE, standard error of the estimate.

### Associations of constitution (height and weight) with organs, tissues and resting energy expenditure

**Figure 1:**
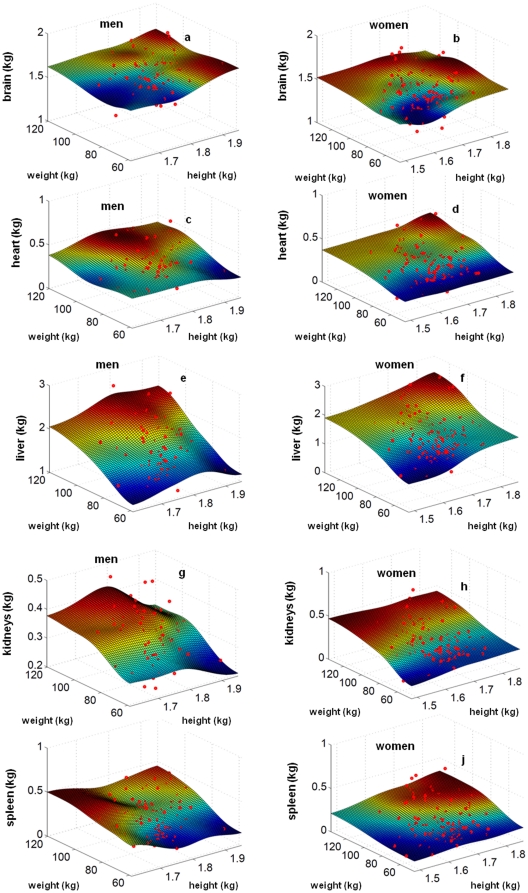
Masses of brain, heart, liver, kidneys and spleen plotted against height and weight for men (a,c,e,g,i) and women (b.d,f,h,j).

In both sexes, there were similar associations between organ and tissue mass, and body weight. Nearly all organs and tissues as well as REE ware inter-correlated with weight and height with non significant correlations between masses of kidneys or spleen and height in men and fat mass or brain mass and height in women, respectively (**Table 2**). Scaling exponents were above 1 in the case of fat mass and spleen whereas weight scaling exponents were between 0.71 and 0.88 in the case of FFM, muscle mass, liver, and kidneys with very low exponents in the cases of brain and heart. Using one-dimensional analogues (comp. methods) these data were used to fit the organ masses to the height and weight (**Fig.1**). Alternatively a functional form using height x weight was used to predict individual organ masses (**Table 2**). When compared with each other there were differences in the a-values but similar data were obtained for the exponents b and c.

**Table 2:** Partial correlation coefficients controlled for age and regression equations between high metabolic rate organs and tissues as well as REE and either body weight or height (n = 262).

	weight (W)		height (H)		weight (W) ×height (H)	
	r	regression	r	regression	r	regression
**FM_DXA_** (kg)	**0.79** **	0.009×W^1.78^	**−0.14** *	48.62×H^−1.42^	**0.84**	0.02×W^2.23^×H^−5.3^
**FFM_DXA_** (kg)	**0.70** **	3.01×W^0.66^	**0.72** **	10.35×H^3.08^	**0.88**	2.03×W^0.48^×H^2.22^
**MM_DXA_** (kg)	**0.70** **	0.93×W^0.76^	**0.70** **	4.18×H^3.40^	**0.85**	0.62×W^0.56^×H^2.41^
**organ mass, MRI**						
** brain** (g)	**0.26** **	0.93×W^0.11^	**0.54** **	0.92×H^0.92^	**0.56**	0.79×W^0.041^×H^0.86^
** heart** (g)	**0.49** **	0.03×W^0.55^	**0.45** **	0.10×H^2.14^	**0.58**	0.024×W^0.42^×H^1.42^
** liver** (g)	**0.70** **	0.10×W^0.62^	**0.47** **	0.57×H^1.92^	**0.73**	0.088×W^0.54^×H^1.04^
** kidneys** (g)	**0.68** **	0.01×W^0.73^	**0.26** **	0.14×H^1.36^	**0.68**	0.012×W^0.72^×H^0.19^
** spleen** (g)	**0.46** **	0.004×W^0.90^	**0.36** **	0.03×H^3.39^	**0.53**	0.003×W^0.73^×H^2.19^
**REE** (MJ/d)	**0.78** **	0.495×W^0.59^	**0.59** **	2.14×H^2.11^	**0.85**	0.39×W^0.49^×H^1.26^
**REE**_adj for weight_ (MJ/d)	**-**	-	**0.55** **	3.60×H^1.17^	**-**	-

*p<0.05.

**p<0.01.

W, weight, H, height; FM, fat mass; FFM, fat-free mass; MM, muscle mass; DXA, Dual X-ray Absorptiometry; MRI, Magnetic Resonance Imaging; REE, resting energy expenditure.

### Association between weight, height and individual organ masses as well as REE

In **Table 3** mean data for the mass of height and weight-adjusted organs are given. Plotting organ mass against weight (x-axis) plus height (z-axis) revealed three dimensional diagrams with different surfaces (**Fig.1**). These surfaces can be used to calculate the percentage mean deviations of measured from mean organ or tissue mass (**Table 4**). When compared with the mass of other organs and tissues there were higher mean deviations and a greater variance of percentage FM. By contrast, the percentage mean deviations between measured and calculated FFM and its individual constituents were small but the variance in data was high.

**Table 3:** Muscle and organ masses adjusted for body height and weight (n = 260) (means±SD, range).

	Means±SD (range)	*SEE*	*n*
**MM_DXA adj for height and weight_** (kg)	27.1±4.7 (15.9–38.4)	*0.29*	*260*
**organ mass, MRI**			
**brain_adj for height and weight_** (g)	1515±157 (30–1890)	*0.009*	*253*
**heart_adj for height and weight_** (g)	338±71 (170–580)	*0.004*	*253*
**liver _adj for height and weight_** (g)	1661±268 (−250–2440)	*0.016*	*257*
**kidneys _adj for height and weight_** (g)	307±52 (180–470)	*0.003*	*250*
**spleen _adj for height and weight_** (g)	256±100 (−60–690)	*0.006*	*257*

MM, muscle mass; DXA, Dual X-ray Absorptiometry; MRI, Magnetic Resonance Imaging, SEE, standard error of the estimate.

**Table 4:** Percentage mean deviations and variances of body composition parameters and REE between measured and calculated values (n = 260) (means, range).

	mean (range)	*SEE*	*n*
**FM_DXA_** (%)	12.7 (−37.6–+130.6)	*1.7*	*260*
**FFM_DXA_** (%)	1.1 (−18.8–19.2)	*0.4*	*260*
**MM_DXA_** (%)	1.4 (−26.6–25.8)	*0.6*	*260*
**organ mass, MRI**			
**brain** (%)	0.5 (−14.2–+20.4)	*0.4*	*252*
**heart** (%)	0.3 (−39.7–+78.0)	*1.1*	*253*
**liver** (%)	1.4 (−30.7–+44.5)	*0.8*	*256*
**kidneys** (%)	2.3 (−36.5–+46.6)	*1.1*	*250*
**spleen** (%)	2.3 (−65.2–+137.9)	*2.0*	*256*
**REE** (%)	1.4 (−25.2–+29.6)	*0.5*	*262*

DXA, Dual X-ray Absorptiometry; FM, fat mass; FFM, fat-free mass; MM, muscle mass; MRI, Magnetic Resonance Imaging; REE, resting energy expenditure, SEE, standard error of the estimate.

Calculation was based on functions as derived from three dimensional plots as shown in Fig. 1.

### Constitution and resting energy expenditure

**Figure 2:**
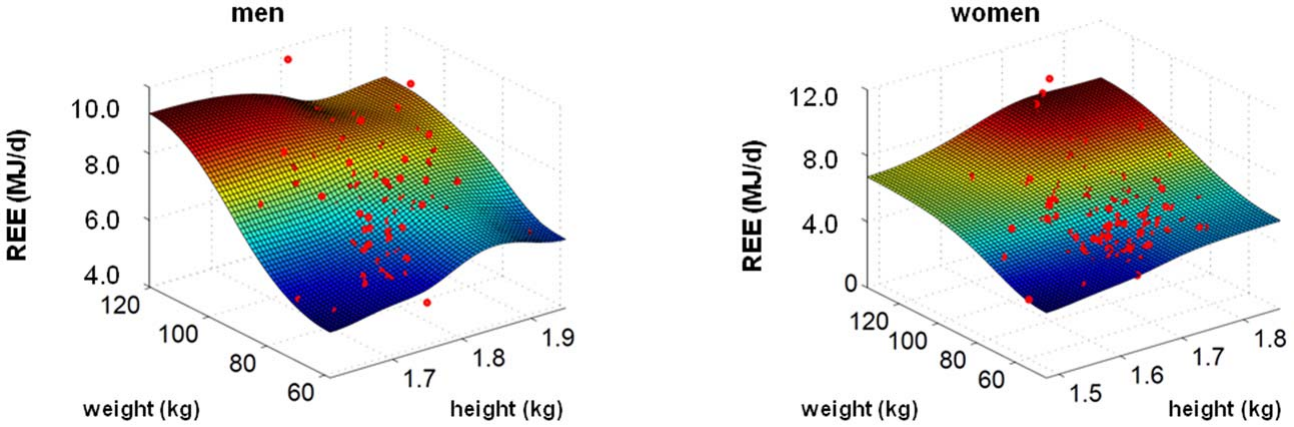
REE plotted against height and weight for men and women.

Plotting REE against weight and height (**Fig.2**) resulted in a 3-dimensional surface reflecting both, increases in REE with weight and height. Partial correlation coefficients between REE and organ mass revealed highest values for muscle and liver mass (**Table 5**). However adjusting organ mass for height and weight reduced most of the associations with REE which still remained significant. When compared to adjustments for height, adjusting for weight had a more pronounced effect on the organ mass-REE association in skeletal muscle, liver, kidneys and spleen. Adjusting brain for either weight or height had only a small effect on the brain-REE relationship. However adjusting for weight plus height reduced the correlation coefficient by about 30%. By contrast to the other organs, adjusting heart mass for weight, height and weight and height had no effect on the association between heart mass and REE. In the case of liver, kidneys and spleen adjusting for height had small or no effects, whereas adjusting for weight had considerable effects on partial correlation coefficients.

**Table 5:** Partial correlation coefficients between muscle and organ masses (non-adjusted and adjusted for constitution) and REE controlled for age (n = 262).

	REE (MJ/d)
**MM_DXA_** (kg)	**0.85** **
**MM_DXA adj for weight_** (kg)	**0.52** **
**MM_DXA adj for height_** (kg)	**0.77** **
**MM_DXA adj for height and weight_** (kg)	**0.50** **
**organ mass, MRI**	
** brain** (g)	**0.54** **
** brain_adj weight_** (g)	**0.46** **
** brain_adj for height_** (g)	**0.49** **
** brain_adj for height and weight_**(g)	**0.46** **
** Heart**(g)	**0.60** **
** heart_adj for weight_** (g)	**0.32** **
** heart_adj for height_** (g)	**0.54** **
** Heart_adj for height and weight_** (g)	**0.31** **
** Liver**(g)	**0.76** **
** liver_adj for weight_** (g)	**0.42** **
** liver_adj for height_** (g)	**0.68** **
** liver_adj for height and weight_** (g)	**0.40** *
** kidneys** (g)	**0.66** **
** kidneys_adj for weight_** (g)	**0.25** **
** kidneys_adj for height_** (g)	**0.61** **
** kidneys_adj for height and weight_** (g)	**0.27** **
** spleen** (g)	**0.53** **
** spleen_adj for weight_** (g)	**0.27** **
** spleen_adj for height_** (g)	**0.47** **
** spleen_adj for height and weight_** (g)	**0.27** **

*p<0.05;

**p<0.01; REE, resting energy expenditure; MM, muscle mass; DXA, Dual X-ray Absorptiometry; MRI, Magnetic Resonance Imaging.

### Regression analyses on the variance of resting energy expenditure

Multiple stepwise regression analysis of the variances of REE adjusted for constitution (dependent variable) in percentage mean deviations between measured and calculated values showed that the constitution-related variances in either FFM (model 1) or kidneys, muscle, brain and liver (model 2) explained up to 35% suggesting that the variances between organ masses and constitution add up to a considerable proportion of the constitution-related variances in REE (**Table 6**).

**Table 6:** Multiple stepwise regression analyses for explained variance of REE independent of constitution in percentage mean deviations between measured and calculates values (n = 262).

Model 1			R^2^	β-Coeff.	SEE
**REE** (%)	1	FFM_DXA_(%)	**0.25**	0.54	7.18
	2	FM_DXA_(%)	**0.26**	0.12	7.13

Model 2			R^2^	β-Coeff.	SEE
**REE** (%)	1	MM_DXA_(%)	**0.19**	0.34	7.41
	2	liver_MRI_(%)	**0.28**	0.27	6.98
	3	brain_MRI_(%)	**0.34**	0.22	6.75
	4	kidneys_MRI_(%)	**0.35**	0.12	6.70

Significant F statistic (p<0,001) for both regression models.

**Model 1:** independent variables [REE (%)]: FM_DXA_(%), FFM_DXA_(%).

**Model 2:** *independent Variables [REE (%)]:* FM_DXA_(%), MM_DXA_(%), brain_MRI_(%), heart_MRI_(%), liver_MRI_(%), kidneys_MRI_(%), spleen_MRI_(%).

In a further multivariate regression analysis, REE ( = dependent variable) was modelled as a linear function of weight, sex, height and age (model1) with a coefficient of determination (R^2^) of 0.76. Replacing weight by FM and FFM (model 2) increased R^2^ to 0.80. Replacing FFM by individual organ masses (model 3) further increased R^2^ to 0.82. Adding constitution-related variances in organ masses (model 4) as further independent variables the final R^2^ was 0.83. The F statistic for the multiple regression analysis showed significant results for all models mentioned. Based on the significance of the F-Test (p<0,001) the increase of R^2^ by stepwise addition of the organ masses and organ mass in relation to constitution to the model is meaningful and has additional explanatory power.

## Discussion

Whole body metabolism is a composite of different metabolic rates in different organs or tissues. FFM is the major determinant of REE, explaining up to 80% of its variance. In addition to FFM, its composition and the proportion of metabolically active mass add a further 4% of explained variance of REE [10], [11]. Each organ and tissue scales differently to body weight and height ([14]; **Table 2**). The present data point out to the idea that in humans the inter-individual variances related to scaling of organ mass to height and weight is a further determinant of the variance in REE. Organ mass has significant associations with REE, with highest correlation coefficients observed for muscle and liver mass. Since there are inter-individual differences in the relation of organ mass to either height and weight, this also adds to the inter-individual variance in REE by up to 43%.

No study has yet considered the independent organ scaling effects of weight and height on REE. Previous authors had linked REE to weight, weight again linked with FFM. Weight also links with height and height is related to REE (through weight). The present study adds relations between weight and height to organs and, thus, to REE. It becomes evident that constitution influences REE through variable organ proportions. Our data analysis is limited to organ mass and could not take into account the specific metabolic rates of individual organs. In addition, we could not address the role of brown adipose tissue (BAT) which may have a considerable effect on the inter- as well as intra-individual variance in REE. PET-CT data on humans have recently shown a prevalence of BAT in the order of 5-10% [15]–[17]. However, the reproducibility estimation was low with only one in eight patients with BAT having positive scans at an additional PET-CT-investigation [17]. So far neither exact BAT mass nor it's specific metabolic activity have been quantified in humans. Preliminary estimates based on animal experiments suggest that 25 or 50 g maximally stimulated BAT may explain up to 20% of energy expenditure in humans [18], [19]. By contrast, interscapular energy expenditure contributes minimally (i.e. <1%) to whole body oxygen consumption in a human study questioning the functional importance of BAT [20], [21]. Anyhow these data suggest that future assessments of functional body composition should include a measure of BAT too.

The present data may also add to discuss recent results related to genome-wide association studies. Both, height as well weight are heritable and polygenetic traits [22], [23]. Although hundreds of loci have been indentified, only a few were associated with both, height and weight (e.g., the melanocortin 4 receptor gene). The genes affecting weight and height also may have an effect on REE. Removal of the effect of genes on constitution may then allow detection of genes affecting REE [24]. Since heritability estimates of REE adjusted for body composition were found to be moderate only (i.e., around 0.30; [25]) the major effect seems to be explained by genes affecting body composition and constitution. The genetics of body composition extends this view. In fact, twin studies suggest that lean body mass is highly heritable (i.e. ranging between 0.56 and 0.60) which was independent of other body measures [26]. Up to now we cannot explain between subject variances in metabolically active components of lean body mass (i.e., organ mass). Since organs scale differently as weight as well as height the “fit” of organ masses within constitution should be considered as a further trait. Thus, besides mass the relation between mass and height is a suitable focus of future genomic research.

The present MRI-data provide evidence for the idea that when compared to the great times of Max Kleiber [1] modern body composition technologies could give considerable insights into the complexity of metabolic rate in humans. In the resting state, we can assume a constant rate of the metabolism of individual organs. Recently, specific metabolic rates of major organs and tissues have been validated across aldulthood with some age adjustments for specific purposes [27]. Since organs differ in their mass as well as their specific metabolic rates they thus differently contribute to metabolic rate of the whole body. The reconstruction of Kleiber's law at the organ-tissue level consisting of five components (i.e., liver, brain, kidneys, heart and remaining tissues resulted in a combined exponent of the product of specific metabolic rates of organs times organ masses to body mass of 0.76 which is close to the exponent of the classic equation [28]. Our present data show that organ masses scale differently to weight and height with scaling exponents of height markedly exceeding the corresponding exponents of weight for fat free mass, skeletal muscle, brain, heart and liver (Table 2). It is tempting to speculate that with larger body mass the contributing effect of height (and thus of the composition of FFM) on metabolic rate increases with metabolically active organs constitute a smaller percentage of body mass.

## Methods

The original study population consisted of 330 healthy, Caucasian volunteers (202 females and 128 males) aged 18 to 78 years with a BMI range of 15.9 to 47.8 kg/m^2^. Participants were recruited from students and staff at the University of Kiel and by notice board postings in local supermarkets and pharmacies. All subjects were non-smokers and took no medication known to influence energy metabolism or body composition. The study protocol was approved by the local ethical committee of the Christian-Albrechts-Universität zu Kiel. Each subject provided informed written consent before participation.

All participants arrived at the metabolic ward of the *Institute of Human Nutrition and Food Science* in the morning at 07.30 h after an overnight fast of >8 h.

### Body composition analysis

A detailed description of the procedures is given in refs 10 and 11.

#### Anthropometrics

Height was measured to the nearest 0.5 cm with subjects in underwear and without shoes (stadiometer Seca, Vogel & Halke, Germany). Weight was assessed by an electronic scale (TANITA, Japan).

#### Dual-energy X-ray absorptiometry (DXA)

Whole body measurement by DXA was performed using a Hologic QDR 4500A, (Hologic Inc., MA, USA). Scans were carried out by a licensed radiological technician. Manufactureś software (version V8.26a:3) was used for the analyses of % fat mass (FM). Appendicular skeletal muscle mass was calculated according to Kim et al. [29].

#### Magnetic resonance imaging (MRI)

The volumes of 4 organs (brain, heart, liver and kidneys) were measured by transversal MRI images. Briefly, scans were obtained by a 1.5T scanner (Magnetom Vision Siemens, Erlangen, Germany). Brain and abdominal organs were examined by a T1-weighted sequence (FLASH) (TR: 177.8 ms (abdominal organs); TR: 170.0 ms (brain); TE: 4.1 ms/echo). ECG-triggered, T2-weighted turbo spin-echo ultrashot scans (HASTE) (TR: 800.0 ms; TE: 43 ms/echo) were used to examine the heart. The slice thickness ranged from 6 mm for brain to 7 mm for the heart and 8 mm the internal organs without interslice gaps. Cross-selectional organ areas were determined manually using a segmentation software (SliceOmatic, version 4.3, TomoVision Inc. Montreal, Canada). Volume data were transformed into organ masses using the following densities: 1.036 g/cm^3^ for brain, 1.060 g/cm^3^ for heart and liver, 1.054 g/cm^3^ for spleen and 1.050 g/cm^3^ for kidneys [30].

### Resting energy expenditure

REE was measured by indirect calorimetry (REE_m_) using a ventilated hood system (Vmax Spectra 29 n; SensorMedics BV, Viasys Healthcare, Bilthoven, Netherlands; software Vmax, version 12-1A). CV for repeated measures of REE in 11 subjects was 5.0% [31]. Calibration of flow and gas analysers was performed immediately before each measurement. Continuous gas exchange measurements were obtained for a minimum of 30 min. The first 15 min of each measurement were discarded. Measurements were performed in a metabolic ward at constant humidity (55%) and room temperature (22°C). REE was from VO_2_ and VCO_2_ calculated according to Weir [32].

### Data analysis

All data are given as means and standard deviations (SD). Statistical analyses were performed using SPSS© for Windows 13.0 (SPSS Inc., Chicago, IL, USA). Differences between gender were analyzed by t-test for independent samples. Pearsońs correlation coefficient was calculated for relationships between variables. Logarithmic regressions were used for adjustments, see Eq. (**). In addition a stepwise multiple regression analysis was performed to explain the effect of body composition (as independent variable either adjusted or non-adjusted for constitution) on variance in REE (dependent variable). Hierachical blocks were entered for tests for change in F-statistics. All tests were 2-tailed and a P-value<0.05 was accepted as the limit of significance. MATLAB© (The MathWorks, Inc., Massachusetts, USA) was used to plot three dimensional graph.

#### Three dimensional data interpolation

In a group with 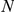 probands, every proband has a height 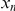 in m, a weight 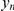 in kg and the measured quantity 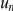 of an organ mass or of REE. We generalize the two-dimensional linear regression to interpret the measured data and to demonstrate the curvature behavior of an interpolating function giving the mean prediction of the measured quantity in the group.

The linear regression of scattered data finds a linear function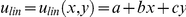 by minimizing the squared error sum
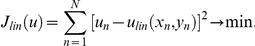



The minimization provides the real values 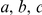 and thus the linear regression function 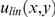 with vanishing curvature.

The generalization of this idea allows the function 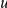 some curvature, and the nonlinear regression function 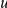 is found by the minimization of a weighted sum of the squared error sum and the total curvature. The objective function now reads
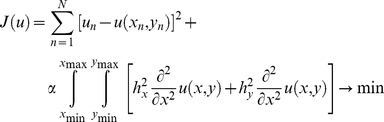
(1) with 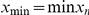, 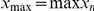 and 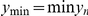, 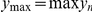 as well as 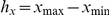 and 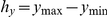. The factors 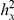
and 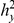 in Eq. (*) scale the second derivatives in the two directions so that there curvatures becomes comparable even if weight and height are measured in different scales.

The weight 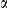 makes a compromise between the two objects under minimization, namely the squared error sum and the total curvature. A small 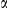 boosts the minimization of the squared error sum whereas a larger 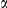 diminishes the curvature of the resulting interpolating function 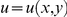. In the limit 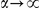, the result of the nonlinear interpolation (*) tends to the linear regression function.

The minimization (*) is computed numerically, so that a large set of values of the function 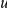 on a rectangular grid is calculated and depicted for different measured quantities in Figures 1 and 2 as a continuous surface. The weight 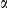 has been chosen large enough, so that strong oscillations of the function 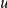 effected by measurement errors or individual deviations of the probands' data are suppressed and the tendency of the curved two-dimensional interpolation 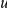 is observable.

The individual deviation of the proband No. 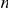
's data from the interpolating function is the quotient 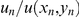 between the actual individual value 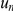 and the prediction 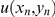 within the group. If the quotient is larger than 1, then the measurement 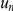 is higher than the mean prediction for the particular height and weight of this proband. In this case the measurement is visible in Figures 1 and 2 as red point above the surface.

The nonlinear approximation quality can be given by the quotient of the mean squared error in relation e.g. to the mean value of the measured data, i.e.
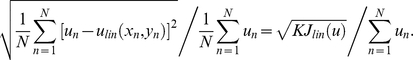



#### Comparison to classical logarithmic regression

The results of the nonlinear regression in Eq. (*) can be compared with the classical logarithmic regression with respect to the power law
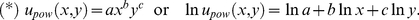
(2)


The logarithmic form of the power law allows a linear regression by minimizing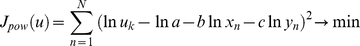 to determine the parameters 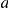, 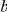 and 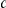.

Table 2 presents the logarithmic regression for the one-dimensional analogues 
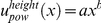
 and 
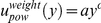
, respectively. The comparison shows that the nonlinear regression reflects the inner behavior of the dependencies of the respective 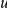 on the height 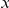 and on the weight 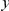, whereas the logarithmic regression determines a lower number of parameters for the data of the entire sample. Alternatively, a two-dimensional analogue term was used as 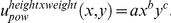


#### REE prediction from organ masses taking into account constitution

Ree was predicted (REEp) from measured organ and tissue masses (m) and organ- and tissue-specific metabolic rates (*Ki* in kJ/kg×d) as taken from the literatur (i.e. 840 for liver, 1008 for brain, 1848 for heart and kidneys, 55 for skeletal muscle, 19 for adipose tissue and 30 for residual mass) (3). REE was then calculated according to 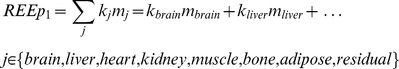



Residual mass includes other tissues such as skeleton, blood, skin, gastrointestinal tract, lung, and spleen. This model assumes a mass-constant *ki*-value. Alternatively, *ki* was expressed as a function of distance between measured organ and tissue masses and organ mass expected from constitution.

Then, REE could be predicted as 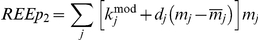 where 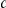 is the mean value of the organ mass indexed by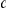. This relation is based on the assumption that the specific metabolic rates depend linearly on the organ masses, i.e. that the energy expenditure of an organ depends quadratically on its mass (which is not true). Again, the relation is linear in 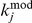 and 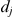, and linear regression can be used to determine these parameters. There are several variants to apply the linear regression for 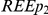: Beside the opportunity to determine all parameters in the same regression, selected values like 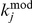 can be used from the literature r from former regressions. Furthermore, a mean value 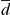 can be regarded instead of several 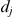. The results of this regression are numerically sensitive. This influences the computations because the data is rather scattered and not large enough to level out the scattering. In particular, the results of the multivariate regression are affected by the selection of the considered organ masses in 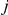.

The computed values 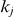 a in the regression of 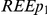 are 1232 kJ/kg (brain), 680 kJ/kg (liver), −370 kJ/kg (heart), 724 kJ/kg (kidney), 80 kJ/kg (muscle), 27 kJ/kg (bone), 14 kJ/kg (adipose) and 47 kJ/kg (residual). The difference to the specific metabolic rates taken from the literature (comp. above) and, in particular, the occurrence of a negative value for the heart are caused by the numerical sensitivity of the multivariate regression. It should be mentioned that the inter-individual differences in body weight and height exceed the corresponding differences in individual organ masses suggesting some limitations of the mathematical approach. Under this disclaimer, we present the results of the further variants of the multivariate regression. A combined determination 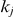 and 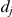 gives 266, 1331, −757, 869, 90, 422, 28, −32 kJ/kg in the above order and 804, −376, 1100, −831, −0.12, −82, −0.35, 2.7 kJ/kg^2^ for values 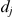. The mean value 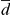 is −0.09 kJ/kg^2^ if the values 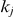 are determined simultaneously and −0.52 kJ/kg^2^ if the values given above are used. Taken together, these values give a first information only. If sound, they would suggest that liver and kidney masses smaller than expected for a given constitution may have higher specific metabolic rates than corresponding organ masses which fit within a given constitution. A valid determination of the values 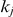 and 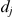 requires a more detailed analysis of the numerical sensitivity and data of a larger sample.

## References

[1] Kleiber M (1932). Body size metabolism.. Hilgardia.

[2] Heymsfield SB, Childers D, Beetsch J, Allison DB, Pietrobelli A (2007). Body size and human energy requirements: reduced mass-specific resting energy expenditure in tall subjects.. J Appl Physiol.

[3] Elia M, Kinney JM, Tucker HN (1992). Organ and Tissue Contribution to Metabolic Rate.. Energy Metabolism: Tissue Determinants and Cellular Corollaries.

[4] Gallagher D, Visser M, Wang Z, Harris T, Pierson RN (1996). Metabolically active component of fat free body mass: influences of age, adiposity, and gender.. Metabolism.

[5] Gallagher D, Belmonte D, Deurenberg P, Wang Z, Krasnow N (1998). Organ-tissue mass measurement allows modeling of REE and metabolically active tissue mass.. Am J Physiol.

[6] Gallagher D, Allen A, Wang Z, Heymsfield SB, Krasnow N (2000). Smaller organ tissue mass in the elderly fails to explain lower resting metabolic rate.. Ann N Y Acad Sci.

[7] Illner K, Brinkmann G, Heller M, Bosy-Westphal A, Muller MJ (2000). Metabolically active components of fat free mass and resting energy expenditure in nonobese adults.. Am J Physiol.

[8] Bosy-Westphal A, Eichhorn C, Kutzner D, Illner K, Heller M (2003). The age-related decline in resting energy expenditure in humans is due to the loss of fat-free mass and to alterations in its metabolically active components.. J Nutr.

[9] Hsu A, Heshka S, Janumala I, Song MY, Horlick M (2003). Larger mass of high-metabolic-rate organs does not explain higher resting energy expenditure in children.. Am J Clin Nutr.

[10] Bosy-Westphal A, Reinecke U, Schloerke T, Illner K, Kutzner D (2004). Effect of organ and tissue masses on resting energy expenditure in underweight, normal weight and obese adults.. Int J Obes.

[11] Later W, Bosy-Westphal A, Hitze B, Kossel E, Glüer C-C (2008). No evidence of mass dependency of specific metabolic rate in healthy humans.. Am J Clin Nutr.

[12] Heymsfield SB, Gallagher D, Mayer L, Beetsch J, Pietrobelli A (2007). Scaling of human body composition to stature: new insights into body mass index.. Am J Clin Nutr.

[13] Heymsfield SB, Chirachariyavej T, Rhyu IJ, Roongpisuthipong C, Heo M (2009). Differences between brain mass and body weight scaling to height: potential mechanism of reduced mass-specific resting energy expenditure of taller adults.. J Appl Physiol.

[14] Glazier DS (2005). Beyond the ‘3/4-power law’: variation in the intra- and interspecific scaling of metabolic rate in animals.. Biol Rev.

[15] Cypress AM, Lehman S, Williams G, Tai I, Rodman D (2009). Identification and importance of Brown Adipose Tissue in Adult Humans.. N Engl J Med.

[16] Saito M, Okamatsu-Ogura Y, Matsushita M, Watanabe K, Yoneshiro T (2009). High incidence of metabolically active brown adipose tissue in healthy adult humans. Effects of cold exposure and adiposity.. Diabetes.

[17] Lee P, Greenfield JR, Ho KKY, Fulham MJ (2010). A critical appraisal of the prevalence and metabolic significance of brown adipose tissue in adult humans.. Am J Physiol.

[18] Rothwell NJ, Stock MJ (1983). Luxuskonsumption, diet-induced thermogenesis and brown fat: the case in favour.. Clin Sci (London).

[19] Rosenbaum M, Leibel R (2010). Adaptive thermogenesis in humans.. Intern J Obes.

[20] Brundin T, Hagenfeldt L, Söderberg R, Wahren J (1987). Blood flow, substrate utilization and heat generation in tissues drained by the azygos vein in man.. Clin Physiol (Oxf.).

[21] Brundin T, Wahren J (1991). Influence of a mixed meal on splanchnic and interscapular energy expenditure in humans.. Am J Physiol.

[22] Walley AJ, Asher JE, Froguel P (2009). The genetic contribution to non-syndromic human obesity.. NATURE Rev Genetics.

[23] Lango AH, Estrada K, Lettre G, Berndt SI, Weedon NM (2010). Hundreds of variants clustered in genomic loci and biological pathways affect human height.. NATURE.

[24] Rice T, Tremblay A, Deriaz O, Perusse L, Bouchard C (1996). A major gene for resting metabolic rate unassociated with body composition: Results from the Quebec Family Study.. Obesity Research.

[25] Bosy-Westphal A, Wolf A, Bührens F, Hitze B, Czech N (2008). Familial influences and obesity-associated metabolic risk factors contribute to the variation in resting energy expnditure: the Kiel Obesity Prevention Study.. Am J Clin Nutr.

[26] Livshits G, Kato BS, Wilson SG, Spector TD (2007). Linkage of genes to total lean body mass in normal women.. J Clin Endocrin Metab.

[27] Wang Z, Ying Z, Bosy-Westphal A, Zhang J, Schautz B (2010). Specific metabolic rates of major organs and tissues across adulthood: evaluation by mechanistic model of resting energy expenditure.. Am J Clin Nutr ajcn.

[28] Wang Z, O'Connor TP, Heshka S, Heymsfield SB (2001). The reconstruction of Kleiber's law at the organ-tissue level.. J Nutr.

[29] Kim J, Wang Z, Heymsfield SB, Baumgartner RN, Gallagher D (2002). Total skeletal mass: Estimation by a new dual-energy-x-ray absorptiometry method.. Am J Clin Nutr.

[30] Duck FA (1990). Physical Properties of Tissue..

[31] Bader N, Bosy-Westphal A, Dilba B, Muller MJ (2005). Intra- and interindividual variability of resting energy expenditure in healthy male subjects - biological and methodological variability of resting energy expenditure.. Br J Nutr.

[32] Weir JB (1949). New methods for calculating metabolic rate with special reference to protein metabolism.. J Physiol.

